# QTL-Seq and Fine-Mapping Analyses Identify QTL and Candidate Genes Controlling Snake-like Pod Surface Trait in Vegetable Cowpea Yardlong Bean

**DOI:** 10.3390/plants14101447

**Published:** 2025-05-12

**Authors:** Khwanruedee Thepphomwong, Makawan Srichan, Artitaya Deeroum, Kularb Laosatit, Prakit Somta

**Affiliations:** 1 Department of Agronomy, Faculty of Agriculture at Kamphaeng Saen, Kasetsart University, Kamphaeng Saen Campus, Nakhon Pathom 73140, Thailand; khwanruedee.th@ku.th (K.T.); artitaya.de@ku.th (A.D.); fagrkal@ku.ac.th (K.L.); 2 Tropical Vegetable Research Center, Department of Horticulture, Faculty of Agriculture at Kamphaeng Saen, Kasetsart University, Kamphaeng Saen Campus, Nakhon Pathom 73140, Thailand; makawan.sr@ku.th

**Keywords:** yardlong bean, pod surface, lignin, QTL, QTL-seq

## Abstract

Yardlong bean is a vegetable type of cowpea grown for fresh and immature pods. “Thua Ngu” is a specialty yardlong bean cultivar with its unique snake-like pod surface and highly crispy pods that may be useful for the breeding of a new cultivar(s). The objectives of this study were to determine the mode of inheritance of the snake-like pod trait and locate the genome region controlling this trait in Thua Ngu. Microscopic observation revealed that the shape, size, and organization of cells of immature and mature pods of Thua Ngu were clearly different from those of “Raya” (normal yardlong bean). Fiber analysis showed that lignin content in immature and mature pods of Thua Ngu was 2.05- and 3.45-fold higher than that in Raya. Segregation analysis using F_2_ and F_2:3_ populations of the cross Thua Ngu × Raya demonstrated that a single gene controls the snake-like pod trait. QTL-seq analysis using the F_2_ population revealed a major locus, *qSlp4.1*, for the snake-like pod trait. Fine-mapping using F_2_ and F_2:3_ populations delimited *qSlp4.1* to a 152.88 Kbp region containing nine genes. Genes with functions involved in cell morphology and/or lignin formation, including *Vigun04g163400*, *Vigun04g163600*, and *Vigun04g163700*, were identified as candidate genes for the snake-like pod trait.

## 1. Introduction

Yardlong bean (*Vigna unguiculata* ssp. *unguiculata* cv.gr. *sesquipedalis*), also known as asparagus bean and string bean, is a vegetable type of cowpea (*Vigna unguiculata* (L.) Walp.), a self-pollinated plant with diploid genome (2*n* = 2× = 22) [1]. It is mainly grown and consumed in Southeast Asia and China for young and tender pods. The pods of yardlong bean are usually 30–60 cm in length, albeit the pods of some cultivars are 70–90 cm in length [1,2]. The pods can be harvested several times, starting about 40–50 days after planting. The pods are consumed in both fresh and cooked forms and are prepared into varieties of dishes, such as salad, soup, and stir fired [3]. It is an economic crop of several countries in Southeast Asia and China, albeit there are no official statistics on the cultivation area and production of this crop. In some countries, such as Thailand, Myanmar, Cambodia, Philippines, and Indonesia, yardlong bean is produced commercially year-round. Pod traits related to eating and cooking qualities and appearance, including pod length, thickness, color, tenderness, firmness, crispiness, surface smoothness, and sweetness, are important for the marketing/commercialization of yardlong bean [3].

Yardlong bean is believed to be selected and evolved from the grain cowpea (*Vigna unguiculata* ssp. *unguiculata* cv.gr. *unguiculata*), possibly in South or Southeast Asia [4]. Due to strong selection in pod characteristics, yardlong bean is clearly genetically different from grain cowpea, and genomic regions associated with traits showing differential selection between yardlong bean and grain cowpea have been identified [5]. Landrace cultivars of crops are important sources of genes for the genetic improvement of new commercial/modern cultivars [6]. Thailand lies in a center of diversity of yardlong bean. “Thua Ngu”, which is translated as “snake bean”, is a local yardlong bean cultivar in southern Thailand and is known by local people in the region for its unique snake-like pod appearance and crispiness. Compared with the pods of normal yardlong bean cultivars, the pod surface of Thua Ngu is rougher, thicker, and much more snake-like. In addition, the pods of Thua Ngu are obviously twisted, while those of normal yardlong bean are relatively straight. The pods of Thua Ngu are popularly prepared into stir-fired dishes. Nonetheless, at present, there has been no report on the genetic basis of the pod trait in Thua Ngu.

Plant breeders always focus on pod-related traits in yardlong bean breeding programs. Nonetheless, there is limited knowledge on genetics and genomics on these traits. Quantitative trait loci (QTLs) controlling pod length, pod tenderness, total soluble solid content, and fiber content have been identified in the yardlong bean [3,7,8]. QTLs controlling pod tenderness, pod length, and fiber content appeared to be the same or closely linked [3,7]. In this study, we aimed to determine the mode of inheritance of the snake-like pod trait and locate the genome region controlling this trait in Thua Ngu. A major quantitative trait locus (QTL) controlling the snake-like pod trait was identified, and candidate genes for the trait were discovered.

## 2. Results

### 2.1. Differences in Pod Appearance and Morphology Between Thua Ngu and Raya

**Figure 1:**
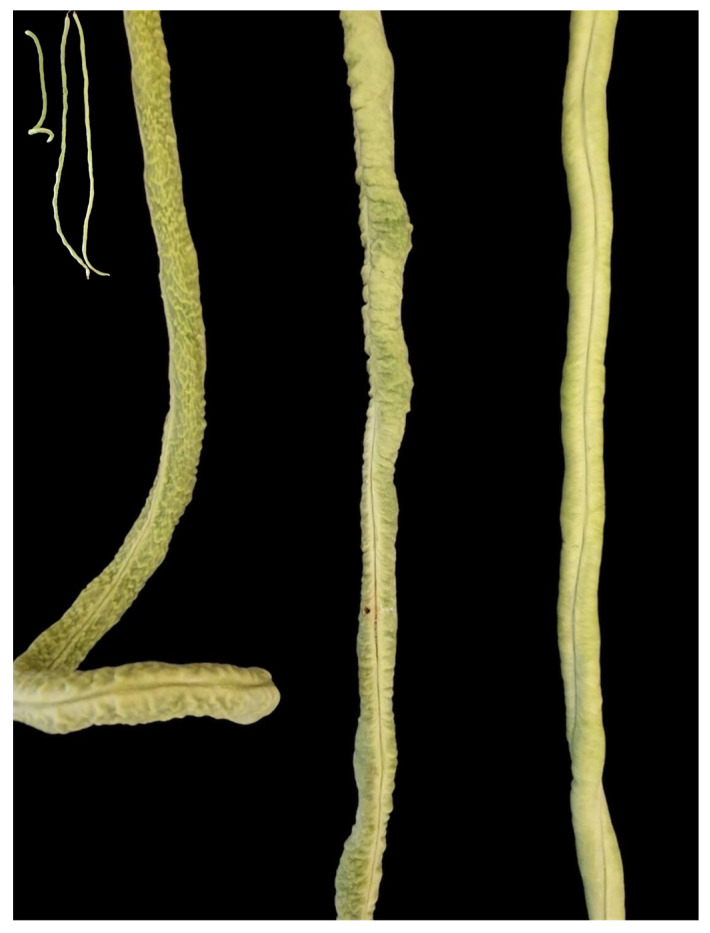
Characteristics of immature pods of Thua Ngu, Raya, and their F_1_ hybrid. Inset shows the whole pods of Thua Ngu (**left**), Raya (**right**), and their F_1_ hybrid (**middle**).

**Figure 2:**
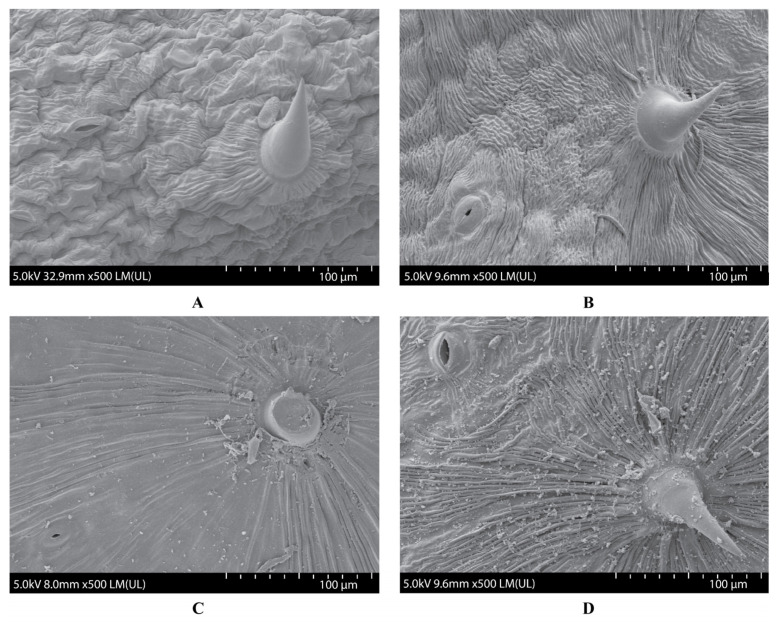
Pod surface structure of immature and mature pods of Thua Ngu and Raya revealed by field-emission scanning electron microscope. Immature pods of Thua Ngu and Raya are shown in (**A**) and (**B**), respectively, while mature pods are shown in (**C**) and (**D**), respectively.

The appearance and morphology of Thua Ngu were compared with Raya. Raya is a normal type and commercial cultivar of yardlong bean. Thua Ngu and Raya expressed contrasting pod appearances. The immature pod surface (marketable pod stage; 8 days after flower blooming) of Thua Ngu was clearly rougher than that of Raya (Figure 1). The observation of the immature pods using a field-emission scanning electron microscope (FE-SEM) revealed that Thua Ngu possessed a large and rough wave-like surface, while Raya possessed a small and fine wave-like surface (Figure 2).

**Figure 3:**
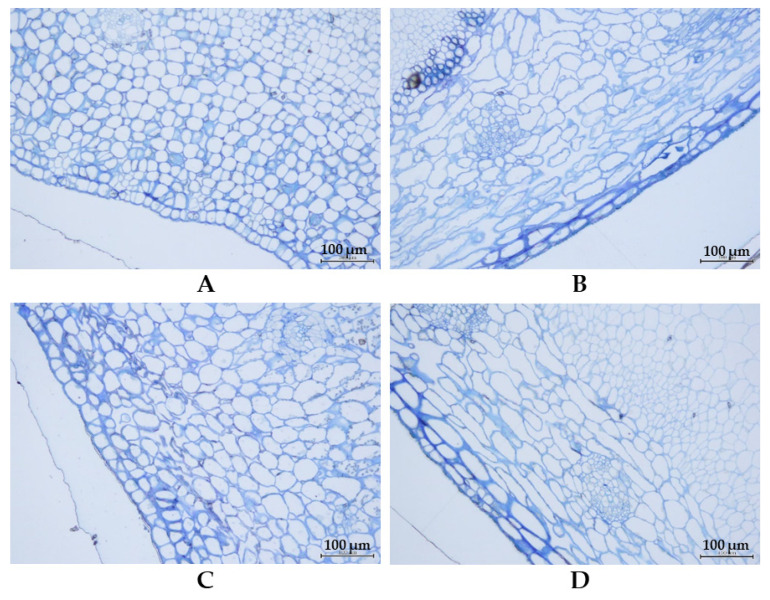
Cell shape, size, and density of immature and mature pods of Thua Ngu and Raya revealed by cross-section microscopy. Immature pods of Thua Ngu and Raya are shown in (**A**) and (**B**), respectively, while mature pods are shown in (**C**) and (**D**), respectively.

The examination of pods in the immature (8 days after flower blooming) and mature stages by cross-section microscopy revealed that in both stages, cells in the pods of Thua Ngu were better organized than those of Raya (Figure 3). In Thua Ngu, the cells appeared to be packed, while in Raya, the cells appeared to be loose. In addition, the shape and size of the cells in the pods of Thua Ngu were different from those of the pods of Raya. In Thua Ngu, the majority of the cells were round or oval in shape, while in Raya, most of the cells were oblong. The differences in cell shape and size were clearly evident in the immature pods.

### 2.2. Differences in Pod Chemical Contents Between Thua Ngu and Raya

Immature and mature pods of Thua Ngu and Raya were analyzed for fiber contents, including cellulose, hemicellulose, and lignin. The results of fiber analysis are summarized in Table 1. In the immature pod stage, Thua Ngu possessed significantly lower cellulose and lignin contents but higher hemicellulose content than Raya. In the mature pod stage, Thua Ngu possessed statistically lower cellulose, hemicellulose, and lignin contents than Raya. However, among these three fibers, the most striking difference between Thua Ngu and Raya was lignin; Thua Ngu had 2.05- and 3.45-fold higher pod lignin content than Raya.

### 2.3. Mendelian Inheritance of Snake-like Pod in F_2_ Population

The inheritance of the snake-like (Ngu-type) pod trait in Thua Ngu was determined in F_2_ and F_2:3_ populations of the cross Thua Ngu × Raya. Chi-square (χ^2^) analysis in the F_2_ population showed that the segregation did not fit the 1 (snake-like pod): 2 (semi-snake-like pod): 1 (normal pod) ratio at the probability level of 0.05, but it fitted the 1:2:1 ratio at the probability level of 0.01 (Table 2). However, χ^2^ analysis in the F_2:3_ population demonstrated that the segregation fitted the 1:2:1 ratio at both probability levels, 0.05 and 0.01 (Table 2). These results suggested that the snake-like pod trait in Thua Ngu is controlled by a single gene, designated as *Snake-like pod* (*Slp*).

### 2.4. QTL-Seq Identified a Major Locus Controlling Snake-like Pod Surface Trait

QTL-seq was carried out to identify the genome region controlling the snake-like pod trait. Four DNA sequencing libraries were prepared: two from the parents (Thua Ngu and Raya) and two from bulked DNA (snake-like pod (SLPD) bulk and normal pod (NPD) bulk). These DNA libraries were whole-genome re-sequenced by using the Illumina NovaSeq 6000 Sequencing System (Illumina, San Diego, CA, USA). Results from the resequencing are summarized in Table 3. The sequencing yielded 157,417,310 reads for Thua Ngu, 141,492,570 reads for Raya, 206,626,290 reads for SLPD-bulk, and 169,252,490 reads for NPD-bulk, which were equivalent to 23.6, 21.2, 21.0, and 25.4 Gb, corresponding to sequencing depths of 30.49, 35.64, 40.47, and 32.92, respectively. The sequence alignment against the cowpea reference genome (Vigna unguiculata 2.1) revealed that the genome coverage rates of these sequence data were 97.39%, 92.08%, 97.18%, and 97.20%, respectively. Read mapping identified a total of 3,545,281 SNPs and 709,233 InDels, with 1,403,307 and 256,049 filtered as high-quality SNPs and InDels, respectively.

**Figure 4:**
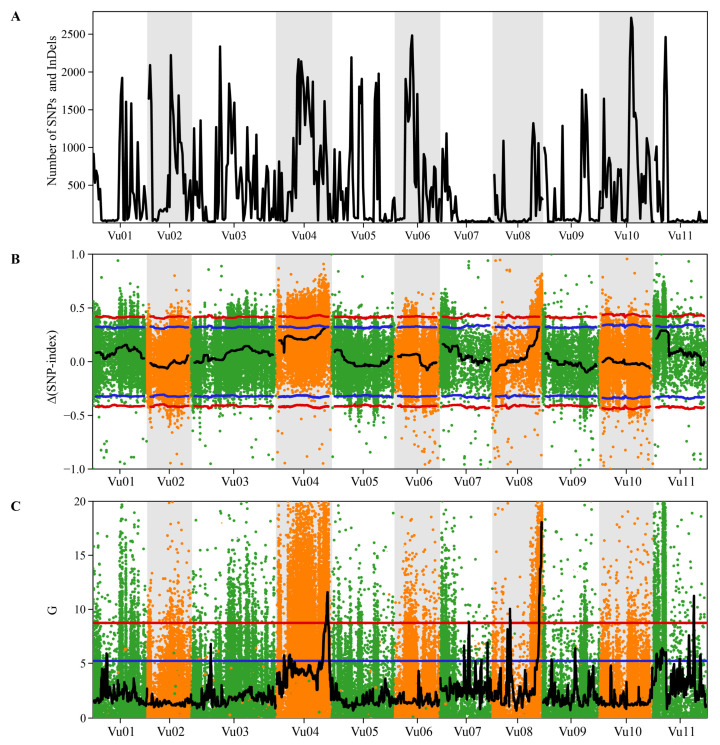
Quantitative trait loci for snake-like pod in yardlong bean identified by QTL-seq. Plots were produced with a 1 Mb sliding window: distribution of single-nucleotide polymorphisms (SNPs) in each window along the mungbean chromosomes (**A**); ∆(SNP-index) with two-sided confidence intervals—95% (red) and 99% (blue) (**B**); and G′-value with two-sided confidence intervals—95% (red) and 99% (blue) (**C**).

QTL-seq analysis using Δ(SNP-index) identified a single QTL for the snake-like pod surface trait (Figure 4 and Table 4). The QTL located on chromosome 4 at the position ranging from 36,200,001 to 42,731,077 with the Δ(SNP-index) value of 0.3253. However, QTL-seq analysis based on the G′-value revealed five QTLs for the snake-like pod surface trait, one each on chromosomes 4, 7, and 11, and two on chromosome 8 (Figure 4 and Table 4). Thus, the QTL-seq analysis by Δ(SNP-index) and G′-statistic pinpointed a single QTL for the snake-like pod surface trait on chromosome 4, designated as *qSpl4.1*.

### 2.5. Fine-Mapping of qSlp4.1

The *qSlp4.1* locus identified by QTL-seq was further investigated by QTL fine-mapping. One hundred and seventy-three new SSR markers were developed from a 400 kb region, 37,999,905 bp to 39,997,744 bp, on chromosome 4 of the cowpea reference genome covering *qSlp4.1* (Supplementary Table S1). Marker screening between Thua Ngu and Raya revealed 77 polymorphic markers (Supplementary Table S1), of which 19 markers were selected and used to analyze the F_2_ and F_2:3_ populations.

**Figure 5:**
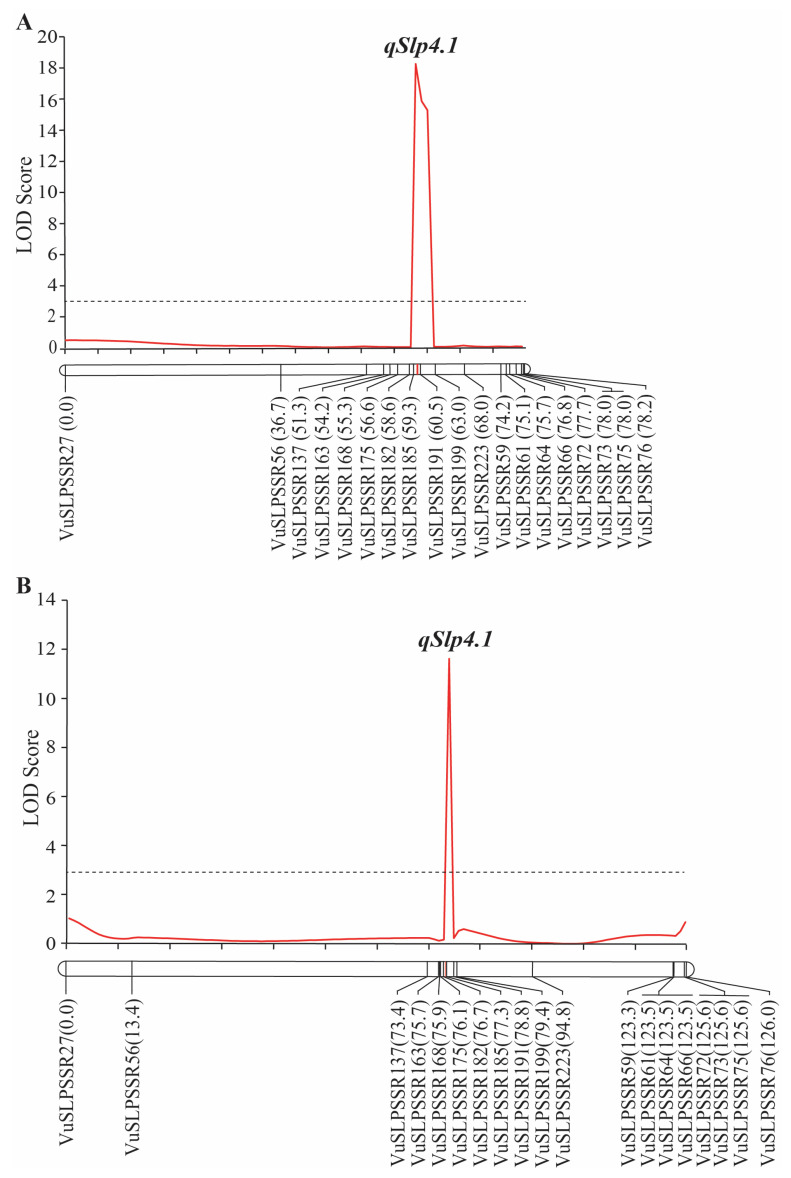
*qSlp4.1* in F_2_ (**A**) and F_2:3_ (**B**) populations of the cross Thua Ngu × Raya detected by inclusive composite interval mapping method. Dotted line represents LOD threshold for QTL.

The linkage map constructed for the F_2_ population was 78.2 cM in total length (Supplementary Figure S1). QTL analysis by the ICIM method revealed that *qSlp4.1* located between the markers VuSLPSSR185 and VuSLPSSR191. *qSlp4-1* explained 31.40% of the pod surface score variation in this population with additive and dominant effects of 0.45 and −0.28, respectively (Table 5 and Figure 5A).

The *qSlp4.1* locus identified by fine-mapping in the F_2_ population was confirmed by using F_2:3_ population. The linkage map constructed for the F_2:3_ population was 126.0 cM in total length (Supplementary Figure S2). ICIM localized *qSlp4.1* to the region between the markers VuSLPSSR182 and VuSLPSSR185. *qSlp4.1* accounted for 19.24% of the pod surface score variation in this population. It showed an additive effect of 0.42 and a dominant effect of −0.06 (Table 5 and Figure 5B).

### 2.6. Candidate Genes

Based on the results of QTL mapping in the F_2_ and F_2:3_ populations, the *qSlp4.1* locus located in the interval region of the markers VuSLPSSR182 and VuSLPSSR191. BLASTN search against the cowpea reference genome revealed that these markers located at the positions 38,728,679 and 38,881,561, respectively. So, the two markers were 152.88 Kbp apart. Based on the cowpea reference genome, there existed nine genes in the 152.88 kbp region (Table 6). Among these, genes are predicted to encode six different proteins, including sugar kinase, MADS-box protein, gibberellin A44 oxidase, carboxylesterase 9-related protein, pentatricopeptide repeat (PPR), and hypothetical protein. Four of the genes encode carboxylesterase 9-related protein.

## 3. Discussion

Local germplasm possessing a specialty/unique trait(s) is a useful genetic resource for the breeding of a new cultivar(s) with high market value and demand [6]. Since yardlong bean is used for its long, crisp, and tender pods in both fresh and processed forms, traits related to appearance, texture, taste, flavor, and nutritive value are important characters for consumer preference and price [3,9]. In this study, we investigated the pod surface, cell wall, and fibers of specialty yardlong bean, Thua Ngu, which possesses a unique immature pod appearance in comparison with the normal yardlong bean, Raya (Figure 1). It appeared that the size, density, and organization of cells and the lignin content in immature pods were clearly different between Thua Ngu and Raya (Figure 2 and Figure 3 and Table 1). Differences in these cell morphologies/properties and lignin content may contribute to snake-like pod appearance and pod crispiness in Thua Ngu. The lignin content in the pods of Thua Ngu was 2.05- and 3.45-fold higher than that in Raya, depending on the pod developmental stage. Lignin is a complex polymer deposited directly in the cell wall of specialized cells. It plays a key role in the appearance and function of vascular pods. It affects plant development by strengthening plant organs and tissue robustness by increasing reinforcement in cell walls [10,11]. It can significantly modify cell morphology, including area, length, width, roundness, circularity, and solidity [12]. Thus, high lignin content may cause high cell density, well-organized/packed cells, and round/oval cell shape in the pods of Thua Ngu.

Segregation analysis suggested that that a single gene controls the snake-like pod phenotype in Thua Ngu (Table 2), and QTL-seq analysis also identified a single QTL, *qSlp4.1*, on cowpea chromosome 4 controlling the snake-like pod phenotype (Figure 4). These results strongly indicate that a single locus controls the snake-like pod phenotype in Thua Ngu. Fine-mapping and bioinformatics analyses revealed nine genes encoding six different annotated proteins as candidate genes for the snake-like pod phenotype (Figure 5 and Table 5 and Table 6). However, based on the function of those genes/proteins, the *Vigun04g163400*, *Vigun04g163600*, and *Vigun04g163700* genes encoding sugar kinase, MADS-box protein, and gibberellin A44 oxidase, respectively, are considered candidate genes for the snake-like pod surface trait in Thua Ngu.

*Vigun04g163600* is predicted to encode a sugar kinase protein that is homolog to the FRUCTOKINASE 1 (FRK1)/FRUCTOKINASE 7 (FRK1) of *Arabidopsis thaliana* L. FRK1 is an enzyme responsible for phosphorylating fructose, converting it into fructose-1-phosphate, a key step in fructose metabolism [13]. Nonetheless, a study in tomato (*Solanum lycopersicum* L.) by gene overexpression and antisense gene suppression illustrated that FRK1 plays an important role in vascular tissue development and that the suppression of this gene results in decreased lignification, leading to distorted xylem vessels and phloem fibers [14]. The study also showed that FRK1 has an effect on cell size and cell wall characteristics in phloem fibers, which are crucial to tissue and plant strength and support [14].

*Vigun04g163600* is predicted to encode a MADS-box protein that is homolog to the AGAMOUS-LIKE6 (AGL6) of *A. thaliana*. MADS-box proteins are transcription factors and play pivotal roles in growth, development, and environmental adaptation, especially flowering time, in plants [15,16]. Nonetheless, a study in *A. thaliana* showed that the *AGAMOUS-LIKE15* (*AGL15*) gene plays a role in lignin biosynthesis through the regulation of the expression of the *PRX17* gene, a peroxidase involved in age-dependent lignified tissue formation, including changes in cell wall properties [17], while a study in chickpea (*Cicer arietinum* L.) demonstrated an association between the *AGAMOUS-like X2* (*CaAGLX2*) gene and pod wall development [18]. In addition, studies showed that ALG6 is a regulator of floral organ identity and spikelet meristem development in wheat [19] and regulates floral organ and meristem identity in rice [20]. Moreover, a recent study in *Phalaenopsis* orchid by virus-induced gene silencing showed that the *ALG6* gene is involved in lignin formation possibly through the regulation of the expression of *VND1*/*MYB46*/*MYB63*/*MYB85* genes [21], which are important regulators of cell wall biosynthesis.

*Vigun04g163700* is predicted to encode gibberellin-A44 oxidase (gibberellin-44 dioxygenase), a key oxidase enzyme in gibberellin biosynthesis that catalyzes the conversion of gibberellin-44 (GA44) into gibberellin-19 (GA19) [22]. GA19 is a precursor to gibberellin-1 (GA1), a bioeffector gibberellin crucial to various growth and developmental processes [23]. Gibberellin (GA) affects plant phenotypes in several aspects through cell growth regulation [24]. For an example, GA-deficient sunflower showed a reduction in leaf area, a thicker lamina, smaller abaxial pavement cells, a stomatal density enhancement, and a guard cell length reduction [25]. However, additional studies including fine-mapping, gene sequencing, and gene expression analysis should be conducted to identify the causal gene controlling the snake-like pod surface trait in Thua Ngu.

## 4. Materials and Methods

### 4.1. Characterization of Pod Morphology

The pod surface and pod wall structure of Ngu and Raya were investigated by using a microscope as per Paopun et al. [26] and Chai et al. [27], respectively. Raya is a commercial and standard type of yardlong bean with normal pod appearance from Thailand. In brief, for the pod surface, fresh and dry pods of each accession were cut into 1 × 1 × 1 mm sections by using a Leica SM2010R Sliding Microtome (Leica Microsystems, Wetzlar, Germany). After that, the samples were treated with 2.5% glutaraldehyde, washed with 0.1 phosphate buffer, and fixed with osmium tetroxide (OsO4). Subsequently, the fixed samples were immersed in liquid nitrogen and dried in a K750X Peltier-Cooled EM Freeze Dryer (Quorum Technologies, East Sussex, UK) for 36 h. After that, the dried samples were coated with gold particles by using an IB-2 ion coater (EIKO Corporation, Tokyo, Japan) and then examined by an SU8020 field-emission scanning electron microscope (FE-SEM) (Hitachi, Tokyo, Japan).

In case of the pod wall structure, cross-sections were obtained. In brief, pods were soaked in sterile water and fixed with 2.5% glutaraldehyde and 4% paraformaldehyde in PBS buffer. After that, the pods were washed with PBS and postfixed in 1% osmium tetroxide, dehydrated in a series of ethanol dilutions, embedded in LR White resin, and polymerized. Cross-sections were cut in the middle of the pod by using a Leica EM UC7 (Leica Microsystems, Wetzlar, Germany).

### 4.2. Determination of Pod Fiber Content

Young and mature pods of pods of Ngu and Raya were analyzed for fiber contents including cellulose, hemicellulose, and lignin, as per the procedures described by Suanum et al. [7] with six plants/replicates. Briefly, young and mature pods harvested from each plant of each cultivar were dried and ground into powder and then sieved through a 1 µm sieve. After that, the powder samples were analyzed for neutral detergent fiber, acid detergent fiber, and acid detergent lignin by using an ANKOM-200 Fiber Analyzer (ANKOM Technology, Macedon, NY, USA). Subsequently, the ANKOM bags containing the residual of the ADF procedure were placed in an ANKOM II Daisy incubator and submerged in 72% H_2_SO_4_. The samples were altered in the incubator, washed by using hot water and acetone, dried, and weighed. Subsequently, the sample bag containing the remaining residual fiber was burnt, and the resultant ash was weighed. Pods of each plant were analyzed twice for fiber contents. The amounts of neutral detergent fiber, acid detergent fiber, acid detergent lignin, and ash were used to calculate cellulose, hemicellulose, and lignin contents.

### 4.3. Population Mapping, Phenotyping of Pod Surface, and DNA Extraction

Two segregating populations, F_2_ and F_2:3_ generations, generated from the crossing of Thua Ngu (female parent) and “Raya” (male parent), were used in this study. Raya is a commercial yardlong bean cultivar with normal pod appearance. The F_2_ population comprised 227 individuals which were grown together with their parents in an experimental field of Kasetsart University, Kamphaeng Saen Campus, Nakhon Pathom, Thailand, from May to August 2021. Eight days after flowering, pods of each plant were harvested for pod surface visual assessment. F_2_ plants having snake-like (Thua Ngu-like) pods, semi-snake-like (F_1_ hybrid-like) pods, and normal (Raya-like) pods (Figure 1) were given scores of 3, 2, and 1, respectively.

The F_2:3_ population comprised 260 individuals. It was derived from the self-pollination of two F_2_ plants showing heterozygosity at the SSR markers VuSLPSSR185 and VuSLPSSR191 flanking the *qPds* QTL detected in the F_2_ population. The F_2:3_ population and its parents were grown from March to June 2023 in the same field used to grow the F_2_ population. The evaluation and scoring of the pod surface in the F_2:3_ population were the same as those described for the F_2_ population.

Total genomic DNA was extracted from young leaves of F_2_, F_2:3_, and parental plants by using the CTAB method [28].

### 4.4. QTL Analysis for Pod Surface Trait by Bulked Segregant Analysis Coupled to Whole-Genome Sequencing (QTL-Seq)

QTL-seq analysis [29] was carried out by using the F_2_ population to identify the QTL controlling pod surface appearance. Two DNA groups of the F_2_ plants expressing contrasting pod surface appearance—normal pod (NPD-bulk) vs. snake-like pod (NGU-bulk)—were constructed. Each group/bulk contained pooled DNA from 25 plants. The DNA of the two bulks and their parents were whole-genome-sequenced by next-generation sequencing. Paired-end sequencing libraries with insert sizes of ~ 350 bp were constructed by using an Illumina TruSeq Library Prep Kit (Illumina, San Diego, CA, USA). The concentration of the libraries was determined by a Qubit^®^ 2.0 fluorometer (Thermo Fisher Scientific, Waltham, MA, USA). The insert size was assessed by using an Agilent^®^ 2100 Bioanalyzer (Agilent Technologies, Santa Clara, CA, USA). Libraries with appropriate insert size and an effective concentration of more than 2 nM were sequenced by using the Illumina NovaSeq 6000 System (Illumina, San Diego, CA, USA). Subsequently, raw paired-end reads were trimmed and quality-controlled by using Trimmomatic [30]. High-quality sequencing reads were aligned against the reference genome of cowpea variety “IT97K-499-35” [31], version 2.1 (Vigna unguiculata v1.2; www.phytozome.net; accessed 15 March 2024), by using the Burrows-Wheeler Alignment tool [32]. Sequence Alignment/Map format files were imported into SAMtools [33]; then, SNPs and short insertion/deletion (InDel) (1–10 bp) were detected by using Genome Analysis Toolkit 4.1.2.0 [34].

QTL-seq analysis was performed by using the R package QTLseqr [35]. SNPs detected with minimum total read depth = 10, maximum total read depth = 500, minimum read depth = 10, and minimum genotype quality (minGQ) = 99 were used in QTL-seq analysis. ∆(SNP-index) [29] and G′-value [36] were calculated by using a sliding window size of 1 Mb, a 95–to-99% confidence interval, and a q-value threshold of 0.01 with 10,000 iterations, while the filter method “∆SNP” was calculated by using a threshold of 0.01 in the G′ analysis.

### 4.5. Development of New DNA Markers and Fine-Mapping of QTL Controlling Pod Surface Trait

Once the QTL controlling the pod surface trait was identified by QTL-seq, the QTL was further investigated by QTL fine-mapping. A genome region of 400 Kb of the reference cowpea genome cultivar IT97K-499-35 [31] covering the location of *qPds* (200 Kb of sequence on the left and right sides of the maximum G′-value position) was searched for simple sequence repeats (SSRs) by using SSRIT software [37]. After that, primers for SSRs were designed by using Primer3 software [38] and screened for polymorphisms between Thua Ngu and Raya by polymerase chain reaction (PCR) and gel electrophoresis, as described by Yundaeng et al. [39]. In brief, PCR was carried out in a total volume of 10 μL containing 5 ng of DNA template, 1 × *Taq* buffer, 2 mM MgCl_2_, 0.2 mM dNTPs, 1 U *Taq* DNA polymerase, and 2.5 μM each of forward and reverse primers. Amplification was performed at 94 °C for 3 min followed by 35 cycles at 94 °C for 30 s, 55 °C for 30 s, 72 °C for 30 s, and 72 °C for 10 min. PCR products were electrophoresed on 5% polyacrylamide gel and visualized by silver staining. Nineteen markers showing clear and polymorphic DNA bands were used to analyze the F_2_ and F_2:3_ populations for QTL mapping.

For each population, a linkage map was constructed by using QTL IciMapping 4.2 [40]. Markers were grouped by using a logarithm of odds (LOD) value of 5.0 and then ordered by using the REcombination Counting and ORDering method [41]. Distances between markers were calculated by using Kosambi’s mapping function [42]. The QTL for CLS resistance was located onto the linkage map by using the inclusive composite interval mapping method (ICIM) [43] by QTL IciMapping 4.2. ICIM was performed at every 0.1 cM with a PIN value of 0.001. An LOD score threshold of 3.0 was used to determine the QTL.

### 4.6. Identification and Sequencing of Candidate Genes

The nucleotide sequences of the SSR markers VuSLPSSR185 and VuSLPSSR191 covering *qSlp4.1* were subjected to BLASTN search against the cowpea genome cultivar IT97K-499-35 [Vigna unguiculata v1.2; accessed 15 June 2024] to determine the physical genome region of *qSlp4.1*. Genes locating within the *qSlp4.1* region were considered candidate genes for the snake-like pod surface trait.

### 4.7. Statistical Analysis

Significant differences in cellulose, hemicellulose, and lignin contents in pods between Thua Ngu and Raya were determined by the *t*-test by using R program 2.14.0 [44]. In the F_2_ and F_2:3_ populations, the numbers of plants exhibiting snake-like pods (pod score = 3), semi-snake-like pods (pod score = 2), and normal pods (pod score = 1) were subjected to segregation analysis by the chi-square test (χ^2^) by using R program 2.14.0 [44].

## Figures and Tables

**Table 1:** Ranges and means of percentage of pod fiber contents in Thua Ngu and Raya.

Type of Fiber	Stage of Pod	Thua Ngu		Raya		*t*-Test	*p*-Value
		Range	Mean ± SD	Range	Mean ± SD		
Cellulose	Immature	9.28–10.88	9.84 ± 0.70	10.17–11.36	10.70 ± 0.53	−2.39	0.0378
	Mature	12.73–21.32	16.61 ± 3.48	18.22–22.53	20.68 ± 1.86	−2.52	0.0304
Hemicellulose	Immature	4.58–5.77	4.94 ± 0.44	3.41–4.67	4.33 ± 0.47	2.35	0.0406
	Mature	7.41–12.73	10.05 ± 1.90	8.90–12.29	11.12 ± 1.49	−1.08	0.3035
Lignin	Immature	0.07–0.64	0.34 ± 0.24	0.35–0.95	0.70 ± 0.25	−2.50	0.0316
	Mature	0.76–1.43	1.10 ± 0.26	2.94–4.44	3.81 ± 0.53	−11.25	0.0005

Data are from six plants/replicates.

**Table 2:** Chi-square (χ^2^) analysis results of the snake-like pod trait in the F_2_ and F_2:3_ populations of the cross Thua Ngu × Raya.

Population	No. of Plants	No. Observed Plants ^1^	No. Expected Plants ^1^	χ^2^ Value	*p*-Value
F_2_	227	56:129:42	1:2:1	5.96	0.05
F_2:3_	260	59:134:67	1:2:1	0.74	0.69

^1^ Snake-like pod: semi-snake-like (F_1_-like) pod: normal pod.

**Table 3:** Statistics of Illumina short-read sequencing for Ngu, Raya, bulked DNA of F_2_ plants with snake-like pods (SLPD-bulk), and bulked DNA of F_2_ plants with normal pods (NPD-bulk).

Sample	Total Reads	Raw Data (Gb)	Clean Reads	Mapping Rate (%)	Genome Coverage (%)	Average Depth
Ngu	157,417,310	23.6	146,209,198	84.5	97.39	30.49
Raya	141,492,570	21.2	130,397,263	84.3	92.08	35.64
SLPD	206,626,290	21.0	191,749,197	89.4	97.18	40.47
NPD	169,252,490	25.4	156,981,684	89.4	97.20	32.92

**Table 4:** QTLs controlling snake-like pod trait in yardlong bean detected by QTL-seq analysis based on ∆(SNP-index) and G′-statistic methods.

Method	Chromosome	Position (bp)	Length (bp)	No. of SNPs and InDels	Peak of Δ(SNP-Index)	Maximum of G-Stat
Δ(SNP-index)	4	36,200,001–42,731,077	6,531,076	3577	0.3253	-
G′-statistic	4	38,000,001–40,500,000	2,499,999	1083	-	11.5729
	7	22,100,001–23,100,000	999,999	22	-	8.7993
	8	12,800,001–14,200,000	1,399,999	17	-	10.0492
		35,300,001–38,363,498	3,063,497	757	-	18.0799
	11	30,700,001–32,000,000	1,299,999	19	-	11.244

**Table 5:** Locations and effects of *qSlp4.1* controlling the snake-like pod surface trait detected by the ICIM method in the F_2_ and F_2:3_ populations of the cross Thua Ngu × Raya.

Population	Location (cM)	Marker Interval	LOD Score	Percentage of Variance Explained by QTL	Additive Effect	Dominant Effect
F_2_	9.0	VuSLPSSR185–VuSLPSSR191	18.19	31.40	0.45	−0.28
F_2:3_	77.00	VuSLPSSR182–VuSLPSSR185	11.63	19.24	0.42	−0.06

**Table 6:** Cowpea genes locating within the interval of markers VuSLPSSR182 and VuSLPSSR191 containing *qSlp4.1*.

Gene	Location on Cowpea Chromosome 4	Encoded Protein	Homologous Gene in *Arabidopsis thaliana*
*Vigun04g163400*	38729477..38732599	Sugar kinase	*AT5G51830* (*FRK1*, *FRUCTOKINASE 1*, *FRK7*, and *FRUCTOKINASE 7*)
*Vigun04g163600*	38737147..38745823	MADS-box protein	*AT2G45650* (*AGL6* and *AGAMOUS-LIKE 6*)
*Vigun04g163700*	38794766..38796717	Gibberellin A44 oxidase	*AT4G25420* (*GA20OX1*)
*Vigun04g163800*	38805392..38806670	Carboxylesterase 9-related protein	*AT5G62180* (*CXE20* and *CARBOXYLESTERASE 20*)
*Vigun04g163900*	38812815..38814262	Carboxylesterase 9-related protein	*AT5G62180* (*CXE20* and *CARBOXYLESTERASE 20*)
*Vigun04g164000*	38820160..38821585	Carboxylesterase 9-related protein	*AT5G62180* (*CXE20* and *CARBOXYLESTERASE 20*)
*Vigun04g164100*	38826706..38830585	Pentatricopeptide repeat	*AT1G80880 (TETRATRICOPEPTIDE REPEAT (TPR)-LIKE SUPERFAMILY PROTEIN*)
*Vigun04g164200*	38830603..38832039	Carboxylesterase 9-related protein	*AT5G62180* (*CXE20* and *CARBOXYLESTERASE 20*)
*Vigun04g164300*	38848168..38852702	Hypothetical protein	*AT5G51800*

## Data Availability

The whole-genome resequencing data generated for Thua Ngu and Raya in this study are available from the National Center for Biotechnology Information (NCBI: https://www.ncbi.nlm.nih.gov) under BioProject numbers PRJNA1244560 and PRJNA1246342, respectively.

## Supplementary Materials

The following supporting information can be downloaded at: https://www.mdpi.com/article/10.3390/plants14101447/s1, Figure S1: Genetic linkage map constructed for F_2_ population of the cross Thua Ngu × Raya. Genetic distance of the markers on the map are shown in unit of centimorgan.; Figure S2; Genetic linkage map constructed for F_2:3_ population of the cross Thua Ngu × Raya. Genetic distance of the markers on the map are shown in unit of centimorgan.; Table S1: Primers used in this study.

## Abbreviations

The following abbreviations are used in this manuscript:

QTL	Quantitative trait locus
ICIM	Inclusive composite interval mapping
FE-SEM	Field-emission scanning electron microscope
SSR	Simple sequence repeat
BLASTN	Basic local alignment search tool for nucleotide sequences
