# Supplementary material for: QTL-Seq and Fine-Mapping Analyses Identify QTL and Candidate Genes Controlling Snake-like Pod Surface Trait in Vegetable Cowpea Yardlong Bean
Source: Plants (Basel). 2025 May 12;14(10):1447. doi: 10.3390/plants14101447 (PMC12114943; doi:10.3390/plants14101447)

Supplementary Figure S2 Genetic linkage map constructed for $F_{2:3}$ population of the cross Thua Ngu x Raya. Genetic distance of the markers on the map are shown in unit of centimorgan.

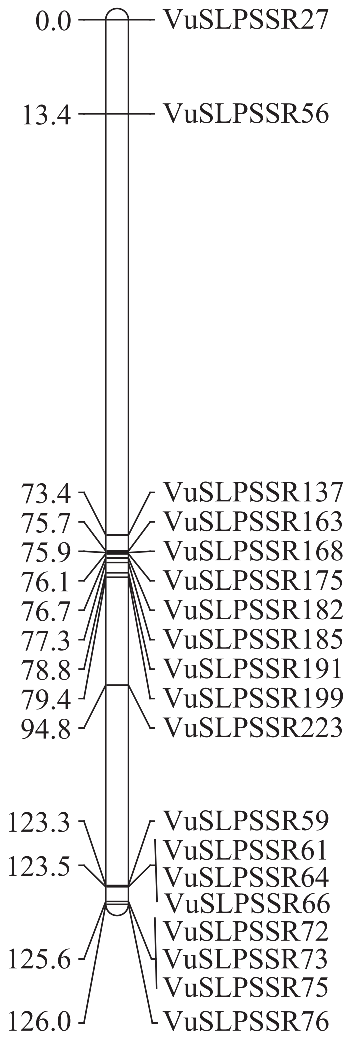

Supplement: Supplementary file 1 [file plants-14-01447-s001.zip › Supplementary Figure S2.pdf]
